# Time-Dependent Proinflammatory Responses Shape Virus Interference during Coinfections of Influenza A Virus and Influenza D Virus

**DOI:** 10.3390/v14020224

**Published:** 2022-01-24

**Authors:** Minhui Guan, Sherry Blackmon, Alicia K. Olivier, Xiaojian Zhang, Liyuan Liu, Amelia Woolums, Mark A. Crenshaw, Shengfa F. Liao, Richard Webby, William Epperson, Xiu-Feng Wan

**Affiliations:** 1 MU Center for Influenza and Emerging Infectious Diseases, University of Missouri, Columbia, MO 65211, USA; mguan@missouri.edu (M.G.); xzm2x@missouri.edu (X.Z.); 2 Department of Molecular Microbiology and Immunology, School of Medicine, University of Missouri, Columbia, MO 65211, USA; 3 Bond Life Sciences Center, University of Missouri, Columbia, MO 65211, USA; 4 Department of Basic Sciences, College of Veterinary Medicine, Mississippi State University, Starkville, MS 39762, USA; sherry.blackmon1@gmail.com (S.B.); ll1257@msstate.edu (L.L.); 5 Department of Pathobiology and Population Medicine, College of Veterinary Medicine, Mississippi State University, Starkville, MS 39762, USA; alicia.olivier@msstate.edu (A.K.O.); amelia.woolums@msstate.edu (A.W.); epperson@cvm.msstate.edu (W.E.); 6 Department of Animal and Dairy Sciences, Mississippi State University, Starkville, MS 39762, USA; MCrenshaw@ads.msstate.edu (M.A.C.); sl1087@msstate.edu (S.F.L.); 7 Department of Infectious Diseases, St. Jude Children’s Research Hospital, Memphis, TN 38105, USA; richard.webby@stjude.org; 8 Department of Electrical Engineering & Computer Science, College of Engineering, University of Missouri, Columbia, MO 65211, USA

**Keywords:** influenza A virus, influenza D virus, co-infection, proinflammatory response, viral interference, swine

## Abstract

Both influenza A virus (IAV) and influenza D virus (IDV) are enzootic in pigs. IAV causes approximately 100% morbidity with low mortality, whereas IDV leads to only mild respiratory diseases in pigs. In this study, we performed a series of coinfection experiments in vitro and in vivo to understand how IAV and IDV interact and cause pathogenesis during coinfection. The results showed that IAV inhibited IDV replication when infecting swine tracheal epithelial cells (STECs) with IAV 24 or 48 h prior to IDV inoculation and that IDV suppressed IAV replication when IDV preceded IAV inoculation by 48 h. Virus interference was not identified during simultaneous IAV/IDV infections or with 6 h between the two viral infections, regardless of their order. The interference pattern at 24 and 48 h correlated with proinflammatory responses induced by the first infection, which, for IDV, was slower than for IAV by about 24 h. The viruses did not interfere with each other if both infected the cells before proinflammatory responses were induced. Coinfection in pigs further demonstrated that IAV interfered with both viral shedding and virus replication of IDV, especially in the upper respiratory tract. Clinically, coinfection of IDV and IAV did not show significant enhancement of disease pathogenesis, compared with the pigs infected with IAV alone. In summary, this study suggests that interference during coinfection of IAV and IDV is primarily due to the proinflammatory response; therefore, it is dependent on the time between infections and the order of infection. This study facilitates our understanding of virus epidemiology and pathogenesis associated with IAV and IDV coinfection.

## 1. Introduction

Influenza viruses are classified into types A, B, C and D according to the genetic and antigenic properties of the nucleoprotein (NP) and matrix 1 (M1) genes [1,2]. Whereas influenza B and C viruses are documented to infect only humans and swine, influenza A virus (IAV) and influenza D virus (IDV) can infect a wide range of hosts. In addition to humans, IAV can infect pigs, horses, dogs, marine mammals (e.g., seals and whales) and a spectrum of avian species, including both wild birds and domestic poultry; IDV can infect domestic and feral swine, cattle, goats, sheep, camelids, buffalo and equids [2,3,4,5,6,7,8] and a low level of human exposure to IDV is also documented [9]. The influenza genome consists of negative-sense, single-stranded, segmented RNA. The genome of IAV contains eight segments, encoding at least 10 or even 14 proteins [10], whereas that of IDV contains seven segments, encoding at least 9 proteins. Based upon the surface glycoproteins, hemagglutinin (HA) and neuraminidase (NA), IAV is further classified into 18 HA and 11 NA types [11,12]. Different from IAV, IDV encodes a hemagglutinin–esterase-fusion (HEF) surface glycoprotein that catalyzes receptor binding, cleavage and membrane fusion [3,13,14], resembling the functions of HA and NA for IAV.

IAV can stimulate both innate and adaptive immune responses with variations that are host-dependent. IAV induces the host’s innate immune response and promotes disease pathogenesis through non-structural (NS1) protein to inhibit TRIM25 ubiquitination, which is required for the activation of retinoic acid-inducible gene I (RIG-I) mediated interferon production [15]. The RIG- I pathway, essential in epithelial cell interferon induction, is induced by preferentially binding viral RNA with a greater affinity for single-stranded RNA without 5′OH or 5′-methylguanosine cap [16]. The presence of RIG-I in ducks (but not in chickens) induces proinflammatory responses and, ultimately, facilitates viral clearance in ducks during IAV infection [17]. In humans, modulating the innate immune response via interferon inhibition often enhances virus production. However, in some cases, the interferon response is too robust and may lead to a “cytokine storm” characterized by overproduction of interferon, leading to upregulation of additional proinflammatory cytokines and excessive infiltration of the tissue by immune cells, leading, in turn, to tissue destruction. The cytokine storm was reported for the 1918 pandemic of the H1N1 virus [18] and H5N1 highly pathogenic avian influenza virus [19,20,21,22].

IDV infection in IDV-seronegative calves causes mild pathogenicity in cattle [23], with a mild respiratory disease with respiratory tract inflammation characterized by multifocal mild tracheal epithelial attenuation and neutrophil infiltration. In field studies, IDV has been associated with the bovine respiratory disease (BRD) complex, a disease of significant economic burden. A previous study had no evidence of cattle coinfected with IDV and *Mannheimia haemolytica,* a pathogen commonly detected in BRD, to have worse clinical scores or lung pathology than animals infected with only *Mannheimia haemolytica* [24]. In animal models, IDV replicated in the upper and lower respiratory tracts of pigs [3,25], guinea pigs [26] and mice [27] and the overall clinical diseases in these animal models caused by a single IDV infection were mild. Nevertheless, the overall role of IDV in pathogenesis, especially during coinfections with other pathogens, including IAV, is still unclear.

Both IAV and IDV are documented to be enzootic in pigs, based on serological evidence from a set of feral swine sera samples collected in the U.S. from 2010 to 2013, where approximately 43% were seropositive for IDV and IAV, suggesting the host–pathogen ecology may include coinfections [25]. In addition, the seroprevalence rate of IDV in IAV-seropositive feral swine was more than twice that observed among IAV-negative feral swine [25]. If IAV and IDV exposures occurred at the same time, there would be a possibility of virus interactions. In domestic swine, several studies showed that domestic swine populations across various geographic regions were seropositive against IDV. In the United States, the seropositive rate for IDV in domestic swine was 9.5% [3]; in France, the seropositive rates within the herd against IDV ranged from 3.3% to 73.3% from 2013 to 2015 [28]; in Italy, seroprevalence increased from 0.6% in 2009 to 11.7% in 2015 [29]; and, in Luxembourg, this rate elevated from 0% in 2012 to 5.9% in 2014–2015 [30]. In China, a qRT-PCR performed on domestic swine samples from asymptomatic animals showed that the IDV-positive rate was also high (28.9%, 13/45) in swine lung samples [7]. Although the sample sizes in some of these studies were small, these data clearly showed IDVs to be enzootic in domestic swine. On the other hand, IAVs are well documented to be enzootic in domestic pig populations worldwide, supporting the high likelihood of IAV and IDV coinfection in domestic pigs.

The objectives of this study are to evaluate the interactions between IAV and IDV during coinfection and to evaluate the pathogenesis during IAV–IDV coinfection in influenza-seronegative pigs. We hypothesized that there would be virus interference among IAV and IDV coinfections and that such interference could be dependent on the time between infections and the order of infections. By using an in vitro system, we compared the proinflammatory responses to IAV and IDV and further correlated these responses with the virus interference patterns and with the factors of infection order and infection time gap. Additionally, we evaluated the clinical pathogenesis of IAV and IDV coinfection using a pig model.

## 2. Materials and Methods

### 2.1. Viruses and Cells

Animals were infected with D/bovine/C00046N/Mississippi/2014 (abbreviated as D/46N) and/or A/swine/Texas/A01104013/2012 (H3N2; abbreviated as sH3N2) isolated from feral swine. D/46N was isolated from sick cattle in Mississippi [4] and propagated in human rectal tumor cells (HRT-18G) (American Type Culture Collection, Manassas, VA, USA), whereas sH3N2 was isolated from feral swine [31] and propagated in MDCK cells (American Type Culture Collection, Manassas, VA, USA). The viruses were propagated in Opti-MEM I Reduced Serum Medium (Thermo Fisher Scientific, Asheville, NC, USA) supplemented with 1 µg/mL of TPCK-trypsin (Gibco, New York, NY, USA) at 37 °C under 5% CO_2._ The predominant exposure for swine in the U.S. is the IAV H3 subtype and strains circulating in feral and domestic swine are antigenically and genetically similar [31,32,33,34]. Because pathogenicity is strain-, dose- and route-dependent, we used swine IAV and IDV stains previously shown by our laboratory to produce successful infection in pigs at 10^6^ TCID_50_/_mL_ by intranasal inoculation [25,35].

To quantify viable cells, we detached the cells with Trypsin-EDTA (Thermo Fisher Scientific, Waltham, MA, USA), mixed them with 0.4% Trypan Blue and quantified them with EVE™ Cell Counting Slides using Cytation 5 (Biotek, Winooski, VT, USA).

### 2.2. Growth Kinetics In Vitro

To determine the replication consequences of the coinfection in cell lines, STECs in 6-well plates were infected with viruses at a multiplicity of infection of 0.001 for IAV and 0.1 for IDV, respectively, based on our pilot study. We performed a series of coinfection experiments in STECs by (1) simultaneous inoculation of IAV and IDV (A + D), (2) sequential inoculations with IAV followed by IDV (A-D groups) with time gaps of 6 (A-D-6h), 24 (A-D-24h) or 48 h (A-D-48h) and (3) sequential inoculations with IDV followed by IAV (D-A groups) with time gaps of 6 h (D-A-6h), 24 (D-A-24h) or 48 h (D-A-48h). The infection groups of IAV (A-single) or IDV (D-single) alone were included as single-infection controls. For the single-infection groups, after absorption for 1 h at 37 °C, the cells were washed with PBS and incubated for 96 h at 37 °C in 5% CO_2_ with Opti-MEM I Reduced Serum Medium (Thermo Fisher Scientific, Waltham, MA, USA) supplemented with 0.25 µg/mL of TPCK treated Trypsin from bovine pancreas (Sigma-Aldrich, St. Louis, MO, USA) and with 100 U/mL of Gibco penicillin-streptomycin (Thermo Fisher Scientific, Waltham, MA, USA). For the multiple-infection groups, the supernatants were removed, washed with PBS and re-infected again at designated hours (6 h, 24 h and 48 h) after the first infection. The supernatants were collected at 24, 48, 72 and 96 h after infection. The RNA copies of each sample were determined with a qRT-PCR. The STECs at 24, 48 and 72 h post-infection were washed, harvested and subjected to total RNA extraction and mRNA expression analyses of cytokines and chemokines.

### 2.3. Quantification of Cytokine and Chemokine Expression

After removing the suppressants, the cells infected with IAV (MOI = 0.001) or IDV (MOI = 0.1) were washed quickly with PBS buffer, treated with lysis buffer and collected for quantification of cytokine and chemokine expression. A RNeasy Mini Kit (QIAGEN, Germantown, MD, USA) was used to extract total RNA from the infected cells following the manufacturing manual. A total cellular RNA of 1 μg was transcribed to cDNA using SuperScript™ III Reverse Transcriptase (Thermo Fisher Scientific, Waltham, MA, USA) with Oligo(dT)20 Primer (Thermo Fisher Scientific, Waltham, MA, USA). The cDNA was used in qPCR using PowerUp™ SYBR^®^ Green Master Mix (Thermo Fisher Scientific, Waltham, MA, USA) and designed primers for specific targets (Table 1). The qPCR amplification mixture contained 7 μL of water, 10 μL of PowerUp™ SYBR^®^ Green Master Mix, 1 μL of each forward and reverse primers (10 μM), 1 μL of cDNA. The parameters of the qPCR were as follows: one cycle at 50 °C for 2 min, one cycle at 95 °C for 2 min, followed by 40 cycles at 95 °C for 1 s and 60 °C for 30 s. The gene expression data were normalized by the house-keeping gene (β-actin). We used the 2^−ΔΔCt^ (Ct is the cycle threshold) methods for qPCR data analysis. Here, ΔΔCt represents ΔCt (sample) ([Ct _gene of interest_ -Ct _housekeeping gene_] of infected cells)—ΔCt (Mock) ([Ct _gene of interest_ -Ct _housekeeping gene_] of uninfected cells). The mean fold change (2^−ΔΔCt^) values of triplicates and standard deviation are represented.

A Porcine IFN-β ELISA Kit (Abcam, Cambridge, MA, USA) was used to quantify the protein expression of IFN-β in supernatants at 24 and 48 hpi from STEC infection following the manufacturing manual.

### 2.4. Viral RNA Extraction

The supernatant from homogenized tissue and the transport media containing the nasal swabs were used for RNA extraction. Viral RNA was extracted using the MagMAX Pathogen RNA/DNA Kit (# 4462359) with a KingFisher™ Flex Purification System (Thermo Fisher Scientific, Waltham, MA, USA) following the manufacturer’s high-throughput purification protocol. Extracted RNA was stored at −80 °C until qRT-PCR could be performed.

### 2.5. Virus Quantification

To quantify the viral copy number in nasal swabs and tissues, a qRT-PCR was performed using standard protocols, primers and probe validated by the CDC for IAV [36] and in-house designed primers and probe to detect IDV (below). Briefly, a qRT-PCR was performed in triplicate by using a TaqMan Fast Virus 1-step Master Mix (Life Technology, Carlsbad, CA) following the manufacturer’s protocol and using 2 µL of RNA template. The samples were amplified using IAV CDC primer and probe set InfA: Forward 5′-GACCRATCCTGTCACCTCTGAC-3′; Reverse 5′-AGGGCATTYTGGACAAA CGTCTA-3′; and Probe 5′-[FAM]-TGCAGTCCTCGCTCA CTGGGCACG-[BHQ]-3′. To detect IDV primer and probe set Forward 5′-ACGCAATGGCACAAGAAC-3′; Reverse 5′-ACCACTATGCTCTCTCCAC-3′; and Probe 5′-[FAM]-AGGAGTTAACCCAATGACCAGGCAAACGA-[BHQ]-3′ were used. The fast-mode amplification protocol was followed, i.e., reverse transcription (1 cycle at 50 °C for 5 min), inactivation (1 cycle at 95 °C for 20 sec), followed by 40 alternating cycles of denaturation at 95 °C for 3 sec and annealing and extension at 60 °C for 1 min.

The viral copies in samples were determined with the standard curve generated by the plasmid containing the target gene segment (IAV M plasmid or IDV M plasmid) cloned into a dual-promoter plasmid vector, pHW2000, as previously described [37,38]. The IDV M plasmid was generously provided by Dr. Richard Webby (St. Jude Children’s Research Hospital, Memphis, TN, USA). The standard curves were plotted by Ct values against viral copy number/mL (nasal swabs) or viral copy number/g (tissue homogenate). Mean Ct values of biological triplicates were recorded and viral copy number concentrations were calculated based on the standard curve constructed across a series of known target concentrations of the plasmid. The data are presented in the figures in log 10 (viral copy number concentration) form.

### 2.6. Growth Kinetics in STECs Pretreated with IFN-Beta

To determine if the IFN-related antiviral-response-inhibited IAV/IDV when coinfected, we pretreated STECs with 0 (mock), 1 or 10 ng/mL of IFN-beta for 16 h before viral infection. The procedures of infection are the same as described in section growth kinetics in vitro. The supernatants were collected at 24, 48 and 72 hpi and titrated by TCID50. The experiment was repeated twice and the representative results are shown.

### 2.7. Animal Study

Animal experiments were conducted under BSL-2 conditions in compliance with protocols approved by the Institutional Animal Care and Use Committee of Mississippi State University. Twenty-five pigs (Large White × Landrace) aged 116–120 days with a mean weight of 43 kg (range of 24–62 kg) were provided by the Mississippi State University Department of Animal and Dairy Sciences (MSU-ADS). The pigs farrowed at MSU-ADS were unvaccinated and biosecurity protocols were in place to limit contact with other animals and personnel with self-reported clinical symptoms of respiratory infection. All pigs were housed together before the study. At 8 dpi and repeated at 0 dpi, all pigs were confirmed serologically negative using an IDEXX Swine Influenza Virus Ab Test kit (Westbrook, ME, USA) and an HI assay against the challenge viruses and other representative influenza viruses. These viruses were chosen to represent the antigenic strains in the vaccine used to vaccinate the sows (Flusure XP^®^ Zoetis, Parsippany-Troy Hills, NJ, USA) at 84 days of gestation as well as seasonal human influenza strains. The pigs were tested seronegative against the following viruses: D/46N (challenge virus), sH3N2 (challenge virus), A/swine/Ohio/09SW96/2009 (H3N2), A/swine/Indiana/13TOSU1154/2013 (H1N1), A/swine/Iowa/15/2013 (H1N1) and human viruses A/Hong Kong/4801/2014 (H3N2) and A/California/04/2009 (H1N1).

At 5 days prior to inoculation, all pigs were transferred to BSL-2 facilities and assigned, by ear tag number, to one of four treatment rooms (12 × 12 feet with negative airflow) as indicated below. Although all pigs were 116–120 days old, their weight range was variable, so they were first stratified into groups of four of similar weights and then randomly assigned to one of four infection treatment groups: A-single group (sH3N2) (*n* = 5), D-single group (D/46N) (*n* = 7), IAV + IDV group (coinfection) (*n* = 7) or negative group (sterile PBS) (*n* = 6). Investigators and animal care personnel were not blinded to the treatment groups and the workflow was controlled; IDV, IAV and coinfection pigs were intranasally infected as follows: the A-single group received 10^6^ TCID_50_/_mL_ of sH3N2 in a volume of 1 mL administered in approximately equal doses to the right and left nostril by syringe; the D-single group received 10^6^ TCID_50_/_mL_ of D/46N using the same method as above; the A + D group received 10^6^ TCID_50_/_mL_ of sH3N2 and 10^6^ TCID_50_/_mL_ of D/46N using the same method as above; and the negative group received sterile PBS using the same method as above. Because pathogenicity is strain-, dose- and route-dependent, we used swine IAV and IDV strains previously shown by our laboratory to produce successful infection in pigs at a smaller dose (10^6^ TCID_50_/_mL_) and by intranasal inoculation [25,35]. The IAV H3 subtype is the predominant influenza virus exposure in feral and domestic swine [32,33,34] and intranasal inoculation simulates a more natural route of infection [39].

To avoid cross-contamination, the pigs in each of the four experimental groups were housed in an individual animal room and all animal rooms had separate airflow. At 5 days prior to inoculation all pigs were transferred to BSL-2 facilities and assigned by ear tag number to one of four treatment rooms. After inoculation, when handling pigs within the same group, 70% ethanol was used to disinfect the surface of the working table as well as PPE. PPE was changed when switching rooms.

During the study, clinical signs, rectal temperatures and nasal swabs were taken daily and whole blood was collected at 0, 3 and 5 dpi. At 5 dpi, all pigs were euthanized and nasal swabs and blood were collected immediately prior to euthanasia. Pigs were necropsied and respiratory tract tissues were collected, including rostral, middle and ethmoid sections of nasal turbinate; soft palate; upper, middle and distal sections of the trachea; bronchus; and one section from each lung lobe (left cranial, left caudal, right cranial, right middle, right accessory and right caudal). The tissues were fixed in 10% buffered formalin and additional sets were frozen at −80 °C.

### 2.8. Clinical Data

To assess clinical signs of influenza infection, before entering the enclosure, pigs were observed from a window for changes in attitude, elevated respiratory rate, cough, dyspnea, nasal or ocular discharge, or conjunctivitis. Rectal temperatures were obtained for all pigs beginning three days before inoculation (−3 dpi) and daily until day 5 of the study (5 dpi). Nasal swabs were collected daily (0–5 dpi) using sterile cotton-tipped applicators and transported in sterile PBS supplemented with PenStrep (1:100 *w*/*v*) on ice to the BSL-2 laboratory where they were aliquoted and stored at −80 °C. Blood samples were taken at 0, 3 and 5 dpi and stored at 4 °C until a complete blood count (CBC) could be performed by the MSU-CVM Diagnostic Lab. A CBC for each pig was obtained with the exception of blood samples that were clotted prior to processing. The samples included one pig from the negative group at 0 dpi, one pig from the D-single group at 0 dpi, two pigs from the A+D group at 3 dpi 5dpi and one pig from the A-single group at 5 dpi.

Respiratory tract tissues were collected at necropsy and frozen at −80 °C until homogenization. Tissues were thawed on ice and a sterile #10 blade and forceps were used to cut and then weigh 1 g of tissue. Tissue samples were placed into prechilled 7 mL autoclaved tubes with prefilled ceramic beads (KT03961-1-302.7; Bertin Instruments, Rockville, MD, USA) and 4 mL of prechilled PBS supplemented with PenStrep (1:100 *w*/*v*). Tissues were homogenized at 8000 × rpm for 20 s for 4 cycles (Precellys^®^ Evolution Homogenizer; Bertin Instruments, Rockville, MD, USA). Sample heating was prevented by incubating the tubes on ice between homogenization cycles. Samples were centrifuged at 15871× *g* (Eppendorf ^®^ 5424; Eppendorf North America, Hauppauge, NY, USA) for 5 min to pellet debris and the supernatant aliquots were stored at −80 °C until RNA extraction and qRT-PCR could be performed.

### 2.9. Histopathological Examination

Respiratory tract tissues were fixed in 10% buffered formalin, paraffin-embedded, sectioned at 5 µm sections and stained with hematoxylin and eosin (H&E) for histopathological examination.

### 2.10. Caspase-3 Stain and Quantification

Tracheal sections were cut at 5 µm on charged slides. The slides were stained with anti-cleaved caspase-3 (Asp175) antibody (Cell Signaling Technology, Boston, MA, USA; Catalog #9661) at a 1:200 dilution following the manufacturer’s protocol for IHC paraffin-embedded tissues. The tissue sections were evaluated at 20× to determine the area with the most abundant staining and then the positive cells from 20 consecutive high-powered fields (40×) were counted. The average number of CC3 positive stained cells from the upper, middle and distal trachea were recorded and analyzed.

### 2.11. Statistical Analyses

A one-way analysis of variance (ANOVA) with repeated measures was used to compare the growth kinetics in cells, with Bonferroni adjustment for multiple comparisons (GraphPad Prism version 8.3.1; GraphPad Software, San Diego, CA, USA). A two-way ANOVA was conducted for the animal study with a replication comparison between the single-infection groups and co-infection group followed by Bonferroni multiple comparisons (GraphPad Prism version 8.3.1; GraphPad Software, San Diego, CA, USA). The CC3 data were log10-transformed and subjected to the Shapiro–Wilk’s test of normality and Brown–Forsythe test for homogeneity of variance. A one-way ANOVA followed by Tukey’s multiple-comparisons test was performed. Differences were considered significant when *p* ≤ 0.05.

## 3. Results

### 3.1. Both IAV and IDV Stimulated Proinflammatory Responses but at a Different Speed

**Figure 1:**
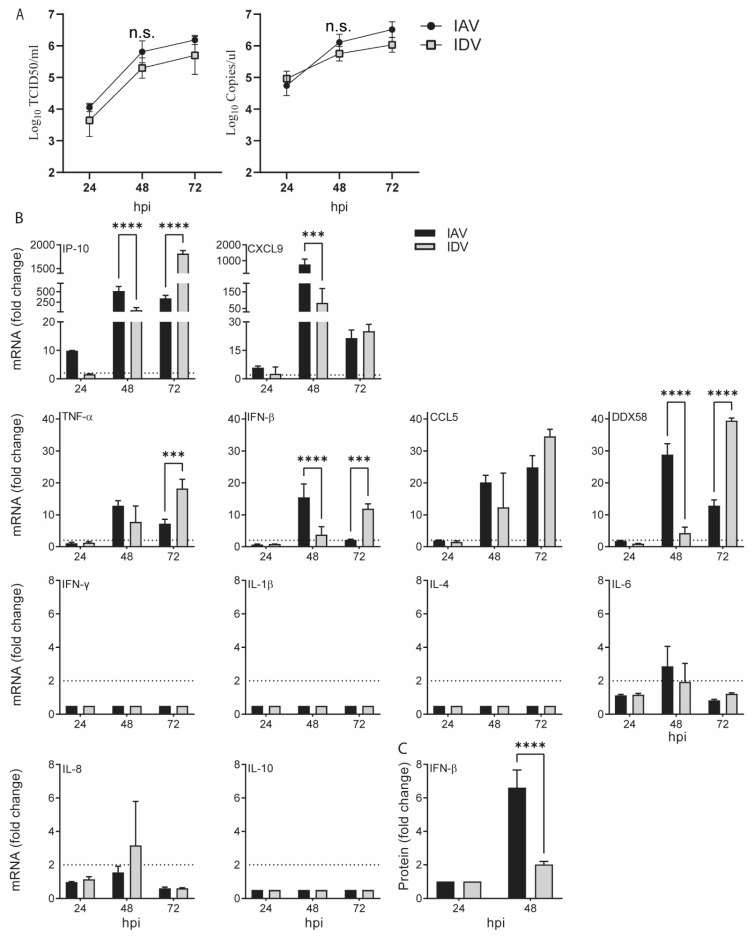
Proinflammatory responses stimulated by IAV and IDV in swine tracheal epithelial primary cells. Proinflammatory responses induced by IAV or IDV alone are shown in black and grey bars, respectively. (**A**) Growth kinetics of IAV and IDV in STECs, titrated by the TCID50 assay (left) and qPCR (right). (**B**) The relative mRNA expressions were quantified by qPCR and normalized by the house-keeping gene across infected and uninfected cells (details in Section 2). The mean values of fold change (2^−ΔΔCt^) for each treatment and standard deviation are represented. (**C**) The protein expression of IFN-β was quantified via ELISA assays. The fold change values of IFN-β’s protein level were calculated (infected samples/negative samples) and are plotted as the y-axis. hpi, hours post inoculation. The dotted line denotes the calculated baselines by the 2^−ΔΔCt^ method. All data collected were obtained from three biological replicates. Significant differences are indicated (* *p* ≤ 0.05, ** *p* < 0.0021, *** *p* < 0.0002, **** *p* < 0.0001) and not significant (*p* > 0.05) differences are denoted by n.s. or not labeled.

To compare the proinflammatory responses stimulated by IAV and IDV, we evaluated both gene and protein expression in swine tracheal epithelial primary cells (STECs) for a set of proinflammatory markers, including type I interferon (IFN-β), type II interferon (IFN-γ), tumor necrosis factor-alpha (TNF-α), DDX58 (retinoic acid-inducible gene I (RIG-I)), interleukin (IL)-1β, IL-4, IL-6, IL8, IL-10, IP-10 (also called CXCL10, interferon-γ-inducible protein 10, previously called IP-10), C–C chemokine ligand 5 (CCL5) and C-X-C Motif Chemokine Ligand 9 (CXCL9) (Table 1), which were reported in IAV and/or IDV infection [27,40,41]. In all in vitro experiments, A/swine/Texas/A01104013/2012(H3N2) (sH3N2) and D/bovine/Mississippi/C00046N/2014 (D/46N) were used and multiplicity of infection (MOI) values of 0.001 and 0.1 were implemented for IAV and IDV, respectively, to ensure the infectious doses and RNA copies for IAV and IDV at the corresponding time points were comparable (Figure 1A).

For IAV infection, the results from the quantitative RT-PCR (qRT-PCR) showed that, compared with those in the negative control, the IP-10 and CXCL9 had the fastest and highest responses, with 9.92 (±0.03; standard deviation)- and 5.89 (±0.87)-fold increases at 24 h post-inoculation (hpi) and with 516.78 (±110.48)- and 768.59 (±330.43)-fold increases at 48 hpi, respectively. The gene expressions of TNF-α, CCL5 and DDX58 were significantly increased at 48 hpi (12.86 ± 1.57, 20.18 ± 2.19 and 28.84 ± 3.33, respectively) and remained elevated at 72 hpi, whereas IFN-β and IL-6 increased at 48 hpi (15.49 ± 4.21 and 2.87 ± 1.19, respectively) but rapidly decreased at 72 hpi. The gene expressions of IFN-γ, IL-4, IL-8, IL-10 and IL-1β were not significantly changed (Figure 1).

Regarding IDV infection, none of the proinflammatory markers we evaluated showed upregulated gene expression at 24 hpi. Similar to IAV infection, the expression of six genes significantly started to increase at 48 hpi (IP-10, 62.20 ± 60.88; CXCL9, 83.68 ± 85.79; TNF-α, 7.78 ± 4.99; IFN-β, 3.79 ± 2.50; CCL5, 12.32 ± 10.76; DDX58, 4.24 ± 1.86) and remained elevated at 72 hpi (Figure 1). Among them, IP-10 and CXCL9 had the highest upregulated expression. The gene expressions of IFN-γ, IL-4, IL-6, IL-8, IL-10 and IL-1β were not significantly affected.

To further validate the proinflammatory responses at the protein level, we quantified IFN-β in cell supernatants harvested at 24 and 48 hpi using the ELISA assay. The results showed that IFN-β was upregulated by 6.61 (±1.05)- and 2.02 (±0.18)-folds for IAV and IDV infections at 48 hpi, respectively, both of which correlated with increased mRNA expression.

In summary, both IAV and IDV stimulated similar proinflammatory responses, including IP-10, CXCL9, TNF-α, CCL5, DDX58, CXCL9 and IFN-β, with a similar level of upregulation; however, the proinflammatory responses to IAV appeared approximately 24 h earlier than IDV.

### 3.2. IAV and IDV Interfered the Replication of One Another during Coinfection

We hypothesized that the proinflammatory responses stimulated by IAV and IDV interfered with virus replication during coinfection; thus, interference would be dependent on the order and time gap of virus infection. To test this hypothesis, we performed a series of coinfection experiments in STECs to simulate simultaneous inoculation of IAV and IDV (A + D), sequential inoculations with IAV followed by IDV (A-D groups) with time gaps of 6 (A-D-6h), 24 (A-D-24h) or 48 h (A-D-48h), or sequential inoculations with IDV followed by IAV (D-A groups) with time gaps of 6 h (D-A-6h), 24 (D-A-24h) or 48 h (D-A-48h).

**Figure 2:**
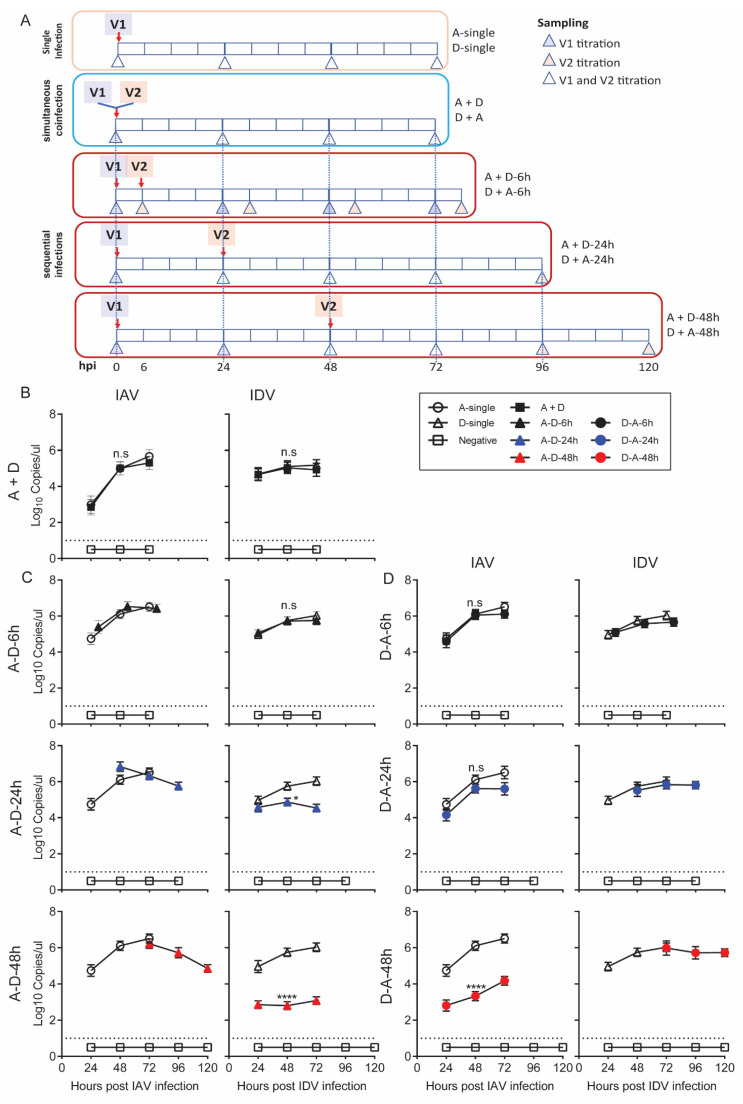
Growth kinetics of coinfecting IAV and IDV in STECs. (A) The experimental design for single infection of IAV or IDV (A-single or D-single), simultaneous coinfection of IAV and IDV (A + D), sequential infections (IAV infection followed by IDV (**A**–**D**) with a time gap of 6 (A-D-6h), 24 (A-D-24h) or 48 (A-D-48h) hours and sequential infections of IDV infection followed by IAV with a time gap of 6 (D-A-6h), 24 (D-A-24h) or 48 (D-A-48h) hours. Both the time points for virus inoculation and sampling are annotated. (B) Growth kinetics of IAV and IDV during simultaneous coinfection. (**C**) Growth kinetics of IAV and IDV during sequential infections of IAV infection followed by IDV. (**D**) Growth kinetics of IAV and IDV during sequential infections of IDV infection followed by IAV. The left panel of each subfigure shows the detection of IAV whereas the right panel shows the detection of IDV. The x-axis of the left panel in each subfigure represents hours after IAV infection whereas the right panel represents hours after IDV infection of the corresponding samples. The dotted line denotes the limit of detection. All data collected were obtained from three biological replicates. Significant differences are indicated (* *p* ≤ 0.05, ** *p* < 0.0021, *** *p* < 0.0002, **** *p* < 0.0001) and not significant (*p* > 0.05) differences are denoted by n.s.

IAV reached titers of 2.99 (±0.38), 5.00 (±0.26) and 5.67 (±0.27) log_10_ copies/µL at 24, 48 and 72 hpi in A-single, respectively; correspondingly, IDV had 4.68 (±0.26), 5.11 (±0.22) and 5.19 (±0.21) log_10_ copies/µL in D-single. In the A+D group, IAV and IDV reached the growth plateau with a titer of 5.01 (±0.27) and 5.03 (±0.22) log_10_ copies/µL at 48 hpi, respectively. Neither of the titers of either IAV or IDV in A+D were statistically significantly different from the corresponding titers in A-single (*p* = 0.842) or D-single (*p* > 0.9999) (Figure 2B).

For the A-D sequential infection groups, at 48 hpi, IDV had 5.72 (± 0.22), 4.87 (± 0.21) and 2.80 (± 0.22) log_10_ copies/ul in A-D-6h, A-D-24h and A-D-48h, respectively. Compared with those in the D-single group, there was a 1.08 (± 0.12)-fold change in A-D-6h, a 7.61 (± 0.29)-fold decrease in A-D-24h and an 882.42 (±16.61)-fold decrease in A-D-48h. The statistical analyses showed that the IDV titers were significantly lower in A-D-24h (*p* = 0.0204) and A-D-48h (*p* < 0.0001) than in D-single, but no significant difference was identified between A-D-6h and D-single (*p* > 0.9999) (Figure 2C). The titers of IAV in all three A-D groups were not statistically different from those in A-single.

For the D-A sequential infection groups, at 48 hpi, the IAV had 6.05 (±0.24), 5.62 (±0.23) and 3.34 (±0.25) log_10_ copies/ul in D-A-6h, D-A-24h and D-A-48h, respectively. Compared with those in the A-single group, there was a 590.44 (±12.39)-fold decrease in the viral titers of IAV at 48 hpi in the D-A-48h groups. The statistical analyses showed that the IAV titers were significantly lower in D-A-48h (*p* < 0.0001) but not affected in D-A-6h (*p* > 0.9999) or D-A-24h (*p* = 0.056) (Figure 2D). The titers of IDV in the A-D groups were not statistically different from those from the D-single group.

Taken together, our results suggest that IAV inhibited IDV replication in STECs when IAV preceded IDV inoculation by 24 or more hours. IDV inhibited IAV replication when IDV preceded IAV inoculation by 48 h. The viral interference correlated with the speed of the proinflammatory responses induced by the first infection, which, for IDV, was slower than for IAV by about 24 h, and the viruses did not interfere when cells were coinfected simultaneously before proinflammatory responses were induced, validating our hypotheses.

### 3.3. IFN-β Pretreatment Suppressed Virus Replication for Both IAV and IDV in STECs

**Figure 3:**
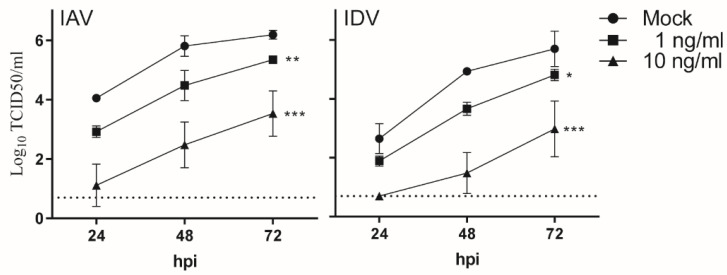
Antagonistic effects of IFN-β on IAV and IDV. STECs were pretreated with a low dose (1 ng/mL) or a high dose (10 ng/mL) of IFN-β for 16 h before inoculating IAV or IDV. A two-way ANOVA analysis was performed to compare the pretreatment group and the mock group (without IFN-β pre-treatment) and significant differences were observed (* *p* ≤ 0.05, ** *p* < 0.0021, *** *p* < 0.0002).

To examine whether elevated expressions can interfere with virus replication for IAV and/or IDV, we selected IFN-β to pretreat the STECs before virus inoculation. The expression level of IFN-β significantly increased with IAV at 48 hpi and with IDV at 72 hpi (Figure 1B). Specifically, we pretreated STECs with a low dose (1 ng/mL) or a high dose (10 ng/mL) of IFN-β for 16 h before inoculating IAV or IDV. Without the IFN-β treatment (mock), both IAV and IDV reached peak titers at 72 hpi, with 6.19 ± 0.12 and 5.70 ± 0.49 log10 TCID50/mL, respectively (Figure 3). When the cells were pre-treated with IFN-β, the viral replication was significantly impaired for both IAV and IDV, with 5.34 ± 0.11 (low dose; *p* = 0.0033; low dose vs. mock) and 3.53 ± 0.62 log10 TCID50/mL (high dose; 0.0002; high dose vs. mock) as IAV peak titers and 4.81 ± 0.16 (low dose; *p* = 0.0157; low dose vs. mock) and 2.98 ± 0.77 (high dose; *p* = 0.0003; high dose vs. mock) log10 TCID50/mL for IDV peak titers. In summary, these results suggest that IFN-β pretreatment suppressed virus replication for both IAV and IDV in STECs and that such interferences were dose-dependent.

### 3.4. Coinfection of IAV and IDV in Pigs Limited Replication of IDV but Not IAV in Upper Respiratory Tracts

To evaluate the pathogenesis during coinfection of IAV and IDV, we simultaneously intranasally inoculated pigs with 10^6^ TCID_50_/_mL_ of sH3N2 and the same amount of D/46N, both of which resulted in effective virus replication and shedding in pigs [25,42]. The single-infection groups (A-single (*n* = 5) or D-single (*n* = 7)) and a control group inoculated with sterile PBS (negative group; *n* = 6) were included as controls.

**Figure 4:**
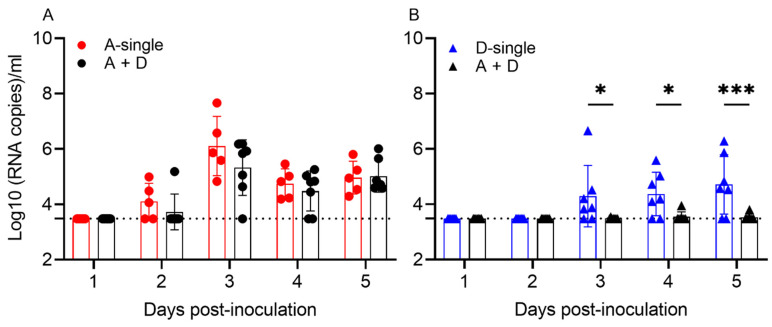
Viral shedding in pigs from the IAV and IDV coinfection experiment. (**A**) Viral titers of IAV. (**B**) Viral titers of IDV. Viral loads in each nasal wash were quantified by qRT-PCR and represented as log_10_ (RNA copies)/mL. Each bar represents the mean values per group and standard deviation. Each data point indicates one sample. The dotted line indicates the limit of detection of 3.48 log_10_ copies/mL sample. Samples from different treatment groups are differentiated by color, i.e., A-single group in red, D-single group in blue and A + D group in black. No IAV was detected in the negative group and D-single group. No IDV was detected in the negative group and A-single group (not shown). Significant differences are indicated (* *p* ≤ 0.05, ** *p* < 0.0021, *** *p* < 0.0002, **** *p* < 0.0001).

The results show that, in the single infections, 5/5 pigs shed IAV and 6/7 pigs shed IDV at 3, 4 and/or 5 dpi, respectively. In A-single, viral shedding peaked at 3 dpi (6.10 log_10_ copies/mL), whereas virus shedding in D-single peaked at 5 dpi (4.72 log_10_ copies/mL). In the coinfection group A + D, 7/7 shed IAV, which peaked at 3 dpi (5.33 log_10_ copies/mL), but only 1/7 pigs shed IDV, which peaked at 4 dpi with low viral copies (3.95 log_10_ copies/mL). To evaluate how coinfection affected viral shedding, we compared viral shedding between A-single and A + D and found that IAV shedding showed no significant difference during the five days (*p* = 0.1262). Of interest, pigs in the coinfection A + D group showed significantly decreased shedding of IDV, compared to D-single (*p* < 0.0001) (Figure 4).

**Figure 5:**
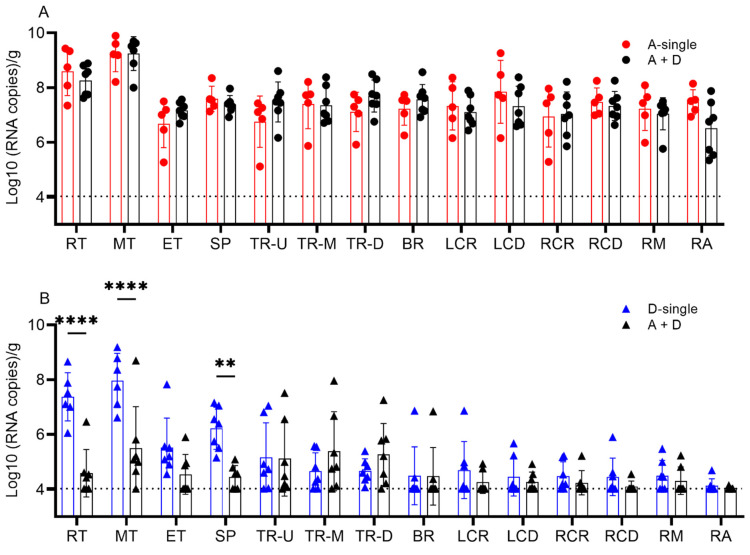
Viral titers in the respiratory tract tissues of pigs from the IAV and IDV coinfection experiment. (**A**) Viral titers of IAV. (**B**) Viral titers of IDV. Viral loads were quantified by qRT-PCR and represented as log_10_ (RNA copies)/g. Each bar represents the mean values per group and standard deviation. Each data point indicates one sample. The dotted line indicates the limit of detection of 4.03 log_10_ copies/g tissue. Samples from different treatment groups are differentiated by colors, i.e., A-single group in red, D-single group in blue and A+D group in black. No IAV was detected in the negative group and D-single group. No IDV was detected in the negative group and A-single group (not shown). A two-way ANOVA analysis was performed to compare the single-infection and coinfection groups and significant differences are indicated (* *p* ≤ 0.05, ** *p* < 0.0021, *** *p* < 0.0002, **** *p* < 0.0001). Abbreviations: rostral turbinate (RT), middle turbinate (MT), ethmoid turbinate (ET), soft palate (SP), upper trachea (TR-U), middle trachea (TR-M), distal trachea (TR-D), bronchus (BR), left cranial lung (LCR), left caudal lung (LCD), right cranial lung (RCR), right caudal lung (RCD), right middle lung (RM) and right accessory lung (RA).

To further evaluate the coinfection on tissue-dependent viral replication, all pigs were euthanized on 5 dpi and the viral load in the tissues of the respiratory tree was quantified. The fourteen respiratory tract tissues were categorized into the following four groups: (1) turbinate (rostral (RT), middle (MT) and ethmoid turbinate (ET)); (2) trachea (upper (TR-U), middle (TR-M) and distal (TR-D)); (3) soft palate (SP); and (4) lower respiratory tract (bronchus (BR); lung left cranial (LCR) and caudal (LCD); right cranial (RCR) and caudal (RCD); and middle (RM) and accessory (RA) lobes). In tissues from both A-single and A+D, all pigs were positive for IAV with a detection limit of 4.03 log_10_ copies/g. There was no significant difference between A-single and A + D for respiratory tissue IAV replication (*p* = 0.8214) (Figure 5A).

All pigs in the groups D-single and A+D were positive for IDV. The IDV copy number was lowest in the lower respiratory tract at 4.46 and 4.25 log_10_ copies/g, for the single and coinfection groups, respectively. In the single-infection IDV group, the IDV copy number was the highest in the turbinate average at 6.96 log_10_ copies/g and the lowest in the trachea at 4.84 log_10_ copies/g (*p* < 0.001) and lower respiratory tract at 4.46 log_10_ copies/g (*p* < 0.001). The differences were also significant between the soft palate (6.23 log_10_ copies/g) and lower respiratory tract (4.46 log_10_ copies/g) (*p* < 0.001) and trachea (4.84 log_10_ copies/g) (*p* = 0.001). In A + D, the IDV copy number was higher in the trachea (5.27 log_10_ copies/g) than in the lower respiratory tract (4.25 log_10_ copies/g) (*p* = 0.032). There was no other difference among tissues in the coinfection group A+D (*p* > 0.137). The IDV copy number was lower in the RT, MT and SP in A+D than in D-single. Specifically, there were significant differences between the RTs with 4.59 vs.7.38 log_10_ copies/g (*p* < 0.0001), MTs with 5.51 vs.7.97 log_10_ copies/g (*p* < 0.0001) and the SPs with 4.45 vs.6.23 log_10_ copies/g (*p* = 0.0018) in A + D and D-single, respectively. The trachea and lower respiratory tract showed no difference in the IDV viral copy number when comparing D-single and A + D (all *p* > 0.05) (Figure 5B).

Taken together, simultaneous co-inoculation of IAV and IDV in pigs significantly reduced viral shedding of IDV and viral replication of IDV in the tissues of the upper respiratory tract, but coinfection did not affect the replication of IAV.

### 3.5. Coinfection of IAV and IDV Virus Did Not Significantly Enhance Disease Pathogenesis

**Figure 6:**
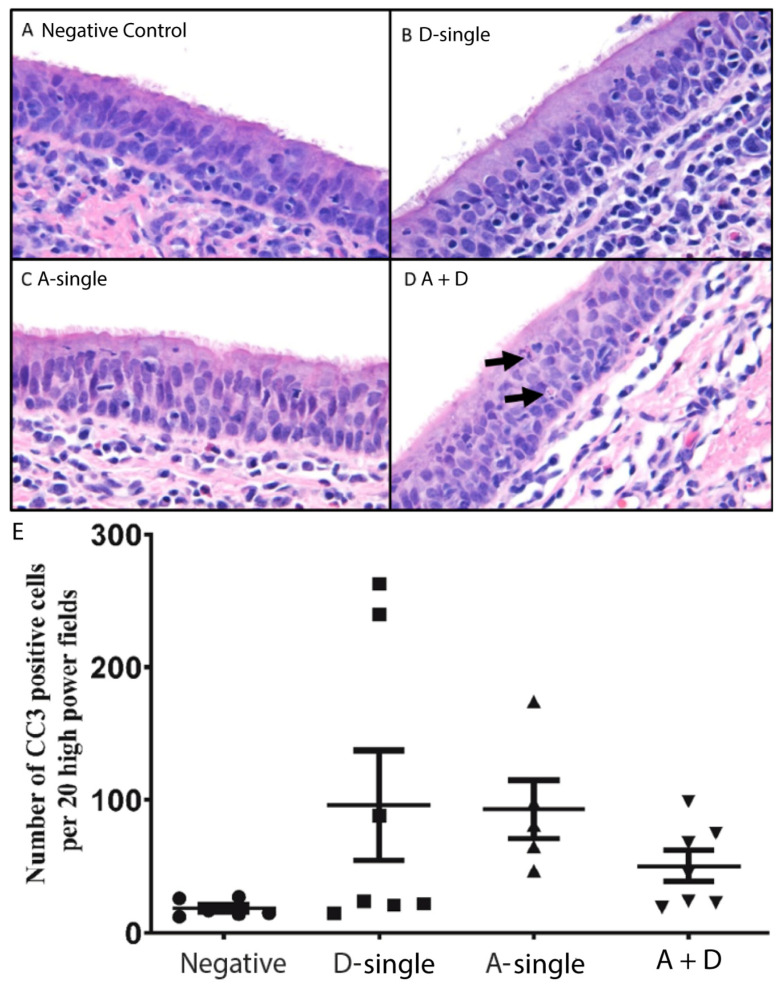
Hematoxylin and eosin staining of tracheas of pigs. (**A**) Negative control (circle), in which pigs were inoculated with sterile PBS; (**B**) D-single (square), in which pigs were inoculated with D/46N alone; (**C**) A-single (up-pointing triangle), in which pigs were inoculated with sH3N2 alone; (**D**) A + D (down-pointing triangle), in which pigs were inoculated simultaneously with D/46N and sH3N2; and (**E**) Cleaved caspase 3 staining in the trachea of pigs. All tracheal tissues showed chronic lymphoplasmacytic inflammation within the mucosa and submucosa. Apoptotic bodies (arrows) were frequently observed (**D**, arrows); however, there was no significant difference among treatment groups and they varied between pigs (**E**).

Pigs in all virally inoculated groups had elevated body temperatures (A-single, D-single and A + D) and lymphopenia. At 3 and 4 dpi, the rectal temperatures were slightly higher in the single-infection groups than the coinfection group; however, there was no statistical difference due to a large variation across pigs in the negative control group. The histologic evaluation of all tissues from the respiratory tract showed no significant differences between treatment groups. This is largely due to chronic inflammatory changes being present in the turbinate, trachea and lung. There was no significant acute inflammatory response in any of the tissues. Chronic tracheal inflammation was characterized by mucosal and submucosal infiltrates of lymphocytes, plasma cells and fewer eosinophils (Figure 6). In some sections of the trachea, apoptotic cells were frequent, but not significantly different among treatment groups (Figure 6D). In all lung sections, including control tissues, the interstitium was moderately thickened due to increased numbers of interstitial macrophages, eosinophils and fewer lymphocytes. There was no evidence of bronchiolar epithelial loss or exudate within the lumen of the airways. The chronic inflammatory-associated pathogenesis changes are thought to be due to the environmental housing and chronic antigenic stimulation.

IAV and influenza B virus, as other viral pathogens, induce apoptosis in vitro and in vivo and apoptosis is associated with the pathogenesis of influenza viruses [43,44]. Cleaved caspase-3 (CC3) is a downstream effector caspase and an important regulator of apoptosis; the activation of Caspase 3 is shown to be essential for efficient influenza virus propagation [45]. To further examine apoptosis-associated pathogenesis among the virally infected groups, we determined, examined and semi-quantified CC3 via immunohistochemistry in the tracheal tissues. In the A-single group, the mean number of CC3 positive cells was 93 (±22 SEM; range of 47–174) which was higher than the mean number of CC3 positive cells in the control group, 19 (±3 SEM; range of 12–27) (*p* ≤ 0.05) (Figure 6E). In the D-single group, the mean number of CC3 positive cells was 96 (± 41 SEM; range of 15–248). Of note, in the D-single group, four pigs had low numbers of CC3 positive cells, but the counts were high for pigs 61 and 71. Interestingly, pig 71 was the only pig in the D-single group that was positive for IDV in all respiratory tract tissues sampled. In the A + D group, the mean was 50 (±12 SEM; range of 19–99). Nevertheless, no statistical significance was found for the number of CC3 positive cells among the A-single, D-single and A + D groups.

Taken together, the coinfection did not lead to significant differences in the clinical signs or pathology compared to the single infections.

## 4. Discussion

The objectives of this study are to evaluate the interactions between IAV and IDV during coinfection and to evaluate the pathogenesis during coinfection in influenza-seronegative pigs. Our in vitro data showed that proinflammatory responses stimulated by IDV at 48 hpi were 24 h delayed compared to proinflammatory responses by IAV, although the expression levels and the associated genes were similar between IAV and IDV infections. Therefore, interference by IAV and IDV depended on both infection order and infection time gap, with no interference observed with simultaneous infection in STECs. In addition to STECs, experiments were also performed in MDCK cells and the results were similar to those observed in STECs (data not shown). An animal challenge with IAV and IDV coinfection showed IDV nasal shedding and viral replication in nasal turbinate and soft palate was decreased during coinfection, suggesting simultaneous coinfection may have antagonistic effects on IDV viral replication in vivo. By examining viral shedding, we found that pigs infected with sH3N2 shed viruses at 2 dpi and peaked at 3 dpi whereas those infected with D/46 shed virus at 3 dpi and peaked at 5 dpi. Thus, we speculate that the fast replication of IAV in pigs may have rapidly stimulated proinflammatory responses in the upper respiratory tracts and, consequently, inhibited the replication of IDV in pigs. Future studies will include the collection of swine bronchoalveolar lavage fluid to evaluate the proinflammatory responses during different days of coinfection. In the tissues across the lower respiratory tract, no statistical difference was identified between IDV titers from D-single and those from A + D groups; however, IDV replication in the lower respiratory tract from the pigs in both the D-single and A+D groups was limited. Of interest, compared with those with IAV viral shedding (Figure 4A), we observed only a low/moderate level of IDV viral shedding in nasal washes in the D-single group but limited in the A + D group (Figure 4B), which were associated with the corresponding viral titers in the upper respiratory tracts (Figure 5B). The low/moderate level of viral shedding in nasal washes in pigs infected with IDV was consistent with those data reported by others [3], as well as in our prior study [25]. Nevertheless, our data support that virus–virus interactions during coinfection are complicated and likely affected by multiple factors, such as the time lag between coinfecting viruses and the rate of virus replication (reviewed in [46]).

Virus–virus interactions, via incredibly diverse mechanisms, are broadly classified by three outcomes, interference, enhancement, or accommodation [46,47], and these interactions can be grouped into 15 mechanisms with three main categories, direct interactions between the viruses, indirect interactions that result from alterations in the host environment and immunological interactions (reviewed by DePalma et al. [47]). The most frequently observed interaction is interference, or when replication of one virus prevents or inhibits the multiplication of the other. Viral interference has also been defined as a state of temporary immunity from infection induced by viral infection [48] and the most common mechanism of viral interference is interferon-mediated. One virus triggers the host interferon response that non-specifically blocks the replication of the other virus. The time of exposure and viral replication are critical factors. On the other hand, viruses may also compete for receptor binding or replication sites, metabolites, or other host supports and this competition can occur between closely related or unrelated viruses. To ensure there are sufficient viable cells prior to the second virus infection, we quantified the viable cells after the first inoculation of IAV or IDV but before the second inoculation. The average viable cell numbers ranged from 1.22 to 2.65 × 10^6^ cells, which correspond to up to 98.55% of the total cells we used in this experiment. A prior study showed that, similar to influenza C viruses, IDVs bound to 9-O-acetylated sialic acid receptors and that IAVs and IDVs showed distinct sialic acid binding preferences in glycan array analyses [49]. The comparable peaking titers in at least A-D-6h and A-D-24h (Figure 2) suggested the receptor competition would play a minor role in shaping the interference between IAV and IDV. On the other hand, our results do suggest that the interactions between IAV and IDV were associated with the proinflammatory responses by either virus, or the inhibitory interference was shown to be bi-directional. Our study also shows that IAV and IDV did not interfere with each other if both viruses were inoculated within a certain time frame, e.g., 24 h when IAV inoculation was followed by IDV inoculation or 48 h when IDV inoculation was followed by IAV inoculation. As described above, such time gaps are more likely associated with the speed of virus replication as well as the proinflammatory responses induced by the first virus.

Several prior studies have detailed IAV to have stimulated proinflammatory responses in humans, various animal models and in various cells [41]. For example, in newborn pig trachea cells, subtype H3N2 swine IAV activated the JAK/STAT and MAPK signaling pathways and stimulated the upregulation of RIG-I, IFN-β, IFN-λ1, Mx1, OAS1, PKR, IL6 and SOCS1 [50]. On the other hand, lung tissues from the mice infected with IDV had minor proinflammatory responses for TLR7, CCL5, IRF3, IL-6, IL-1β and IFN-γ at 1 dpi [27], of which CCL5 had the highest responses. Of interest, although IAV can lead to much higher morbidity in pigs than IDV, our in vitro study using STECs shows both viruses could induce a similar level of proinflammatory responses with the same set of genes, including IP-10, CCL5, CXCL9, TNF-α and IFN-β. Several markers evaluated, IL-4, IL-6, IL-8, IL-10 and IL-1β, had minimal or limited expression. These results indicate that IDV and IAV may share similar signaling pathways, such as JAK/STAT and MAPK, during proinflammatory immune responses.

Apoptosis, or programmed cell death in the absence of inflammation, is an energy-dependent, caspase-mediated biochemical mechanism characterized morphologically by cytoplasmic and nuclear condensation, chromatin cleavage, apoptotic bodies, maintenance of an intact plasma membrane and exposure of surface molecules targeting phagocytosis, as well as efficient removal of the cell and its contents [51,52,53,54]. Activation of CC3 has been shown to be essential for efficient influenza virus propagation [45]. The induction of apoptosis and subsequent phagocytosis of infected cells is also one of the antiviral mechanisms [55,56]. In this study, we evaluated CC3 expression in the trachea, which showed that the average number of CC3-positive cells was higher in the trachea from all pig treatment groups than those from the negative control pig, indicating that the virus caused apoptosis in the infected pigs (Figure 6E). Among the treatment groups, it was interesting that the mean of CC3 -positive cells in either A-single or D-single groups were higher than those in the A + D group; however, no statistical significance was found among the three treatment groups. The results support that coinfection pigs did not have increased pathogenesis compared with either IAV or IDV single-infection pigs.

One limitation of this study is that only a single dose for each virus was used both in vitro and in vivo experiments. Because the level of proinflammatory responses correlates with virus replication, we used two different MOIs to ensure that the quantities of IAV and IDV were comparable in our in vitro experiment (Figure 1A). However, in the animal experiment, we implemented one infection scenario with the same infectious doses for IAV and IDV, which had previously been demonstrated to result in effective infections in swine [25,57]. Nevertheless, as mentioned above in the discussion, the faster virus replication of IAV than IDV can partially explain the interference observed in the simultaneous inoculation in animal experiments but not in the simultaneous inoculation in the STECs. Additional experiments need to further evaluate alternative doses for inoculation, which may affect the interference patterns between two viruses. Future directions could also feasibly test a sequential infection time course in the pig model and tissue-dependent primary proinflammatory cytokines could aid in further understanding the tissue-dependent virus interference in pigs. An additional limitation is that we were not able to determine which specific proinflammatory marker(s) would play a major role in the overall antagonistic effects and whether the predominant proinflammatory markers causing antagonistic effects may differ between IAV and IDV. As a future study, gene-specific RNA interference could be used to test the antagonistic effects of each of these proinflammatory markers for both IAV and IDV, including IFN-β, which suppressed virus replication for both IAV and IDV in STECs with cell pretreatment (Figure 3).

In summary, this study suggests that both IAV and IDV can interfere with the replication of one another by stimulating proinflammatory responses; however, the proinflammatory response to IDV was slower than that to IAV by about 24 h. The mechanism of viral interference appeared to occur via proinflammatory responses and not through viral binding or replication. Coinfection of IDV and IAV in pigs did not show enhanced pathogenesis, compared with those infected only with IAV. This study facilitates our understanding of virus epidemiology and pathogenesis associated with IAV and IDV coinfection.

## Figures and Tables

**Table 1:** Primers used to quantify mRNA expression of swine-specific proinflammatory markers in STECs.

Gene	Forward Primer (5′-3′)	Reverse Primer (5′-3′)
β-actin	GACATCCGCAAGGACCTCTA	ACACGGAGTACTTGCGCTCT
IP-10	GTCGAAGGCCATCAAGAATTTAC	GGCAGAGGTAGATTCTCTCCG
CXCL9	GAGGAATGGACGTTGTTCCTGC	GGGTTTAGACATGTTTGATCCCC
TNF-α	TGCCTACTGCACTTCGAGGTTATC	GTGGGCGACGGGCTTATCTG
IFN-β	AGTTGCCTGGGACTCCTCAA	CCTCAGGGACCTCAAAGTTCAT
IFN-γ	CGATCCTAAAGGACTATTTTAATGCAA	TTTTGTCACTCTCCTCTTTCCAAT
DDX58	CGATGAGGTGCAGCATATTCAGGC	GGAACTGGAGAAAAAGTGATGCAGCC
CCL5	CCCCATATGCCTCGGACACCACA	GTTGGCACACACCTGGCGGTTC
IL-1β	AATTCGAGTCTGCCCTGTACCC	GCCAAGATATAACCGACTTCACCA
IL-4	GGACACAAGTGCGACATCA	GCACGTGTGGTGTCTGTA
IL-6	TGGCTACTGCCTTCCCTACC	CAGAGATTTTGCCGAGGATG
IL-8	TTCGATGCCAGTGCATAAATA	CTGTACAACCTTCTGCACCCA
IL-10	AGCCAGCATTAAGTCTGAGAA	CCTCTCTTGGAGCTTGCTAA
